# Better Few than Hungry: Flexible Feeding Ecology of Collared Lemurs *Eulemur collaris* in Littoral Forest Fragments

**DOI:** 10.1371/journal.pone.0019807

**Published:** 2011-05-19

**Authors:** Giuseppe Donati, Kristina Kesch, Kelard Ndremifidy, Stacey L. Schmidt, Jean-Baptiste Ramanamanjato, Silvana M. Borgognini-Tarli, Joerg U. Ganzhorn

**Affiliations:** 1 Department of Anthropology and Geography, Oxford Brookes University, Oxford, United Kingdom; 2 Department of Biology, University of Pisa, Pisa, Italy; 3 Department of Animal Ecology and Conservation, University of Hamburg, Hamburg, Germany; 4 Département Biologie Animale, Université d'Antananarivo, Antananarivo, Madagascar; 5 Colorado Division of Wildlife, University of Colorado, Fort Collins, Colorado, United States of America; 6 QIT Madagascar Minerals, Rio Tinto, Tolagnaro, Madagascar; Texas A&M University, United States of America

## Abstract

**Background:**

Frugivorous primates are known to encounter many problems to cope with habitat degradation, due to the fluctuating spatial and temporal distribution of their food resources. Since lemur communities evolved strategies to deal with periods of food scarcity, these primates are expected to be naturally adapted to fluctuating ecological conditions and to tolerate a certain degree of habitat changes. However, behavioral and ecological strategies adopted by frugivorous lemurs to survive in secondary habitats have been little investigated. Here, we compared the behavioral ecology of collared lemurs (*Eulemur collaris*) in a degraded fragment of littoral forest of south-east Madagascar, Mandena, with that of their conspecifics in a more intact habitat, Sainte Luce.

**Methodology/Principal Findings:**

Lemur groups in Mandena and in Sainte Luce were censused in 2004/2007 and in 2000, respectively. Data were collected via instantaneous sampling on five lemur groups totaling 1,698 observation hours. The Shannon index was used to determine dietary diversity and nutritional analyses were conducted to assess food quality. All feeding trees were identified and measured, and ranging areas determined via the minimum convex polygon. In the degraded area lemurs were able to modify several aspects of their feeding strategies by decreasing group size and by increasing feeding time, ranging areas, and number of feeding trees. The above strategies were apparently able to counteract a clear reduction in both food quality and size of feeding trees.

**Conclusions/Significance:**

Our findings indicate that collared lemurs in littoral forest fragments modified their behavior to cope with the pressures of fluctuating resource availability. The observed flexibility is likely to be an adaptation to Malagasy rainforests, which are known to undergo periods of fruit scarcity and low productivity. These results should be carefully considered when relocating lemurs or when selecting suitable areas for their conservation.

## Introduction

One of the imperative goals of conservation biology is to determine how animals react to habitat degradation and fragmentation. This knowledge is particularly urgent for forest dwelling primates because of the alarming rate of habitat alteration and the scarce ability of most species to move between forest fragments [1]. Habitat loss and fragmentation can alter both quantity and quality of food resources available to primates [2]–[5]. Logging affects density, size, and distribution of plant species in forest fragments [6], changing the availability of preferred resources for primates, while edge effects result in high mortality of primary forest trees [7]–[9]. However, the primate response to habitat degradation seems to vary depending on species and forest type and no clear generalizations emerge [10], [11].

As a general rule, habitat degradation seems to affect to a lesser extent folivorous primates, since secondary growth may produce higher food quality, i.e. leaves with higher protein and lower fiber content, compared with those found in mature forests [12]–[15]. By contrast, frugivorous primates encounter more problems, due to the fluctuating spatial and temporal distribution of fruiting trees, the need to obtain proteins and minerals from alternative food, and larger home range requirements [16]–[18]. Since frugivorous primates are important seed dispersers and therefore fundamental to catalyze the regeneration of degraded habitats, this vulnerability has major implications for the maintenance of forest diversity [13], . According to evolutionary life history analyses, animals have the option to optimize their energy budget by either minimizing the time spent on food intake (time minimizers) or maximizing the energy intake at the expense of time requirements (energy maximizers) [22]. Different primate species adopt one of the two strategies even though there seem to be some flexibility [23].

Although Madagascar is experiencing a dramatic habitat loss, how lemurs react to forest degradation and logging is not yet clear [24], [25]. Similar to other primates, frugivorous lemurs seem to be particularly vulnerable [26]. This disadvantage is amplified by the unpredictability of fruiting patterns which characterizes the island's environment [27]–[29]. Frugivorous ruffed lemurs, *Varecia* species, for example, are the first to disappear when forest are logged ([30] but see [31]). Also, frugivorous rainforest brown lemurs, *Eulemur fulvus rufus*, are forced to migrate during lean seasons in order to find fruits and meet their energy requirements [26], [32]. On the other hand, some flexibility has been observed and a number of mainly frugivorous lemurs may switch to lower quality foods during lean periods (*E. f. rufus* [33], *Lemur catta* [34], *V. variegata* [35]), modify their activity and ranging patterns (*L. catta* [36], *E. macaco flavifrons* [37], *E. collaris* [38]–[39]), or use food patches of different size and split into subgroups (*E. f. fulvus* [40], *V. rubra* [41]–[42], *V. variegata* [43], *Propithecus diadema* [44]). Thus, adaptations to fluctuating ecological conditions may have potentially selected for the ability of lemurs to cope with degraded habitats. However, the question about the limits of tolerance of frugivorous lemurs to secondary and/or degraded habitats and about which strategies they use to deal with these conditions remain unresolved [25], [45]. Moreover, specific nutritional analyses comparing food items selected in degraded/logged versus intact habitats to assess diet quality have been rarely carried out in Madagascar [13].

The littoral forest of southern Madagascar offers an excellent opportunity to test the flexibility of frugivorous lemurs to degraded habitats. In 2000 the entire population of collared lemurs, *E. collaris*, of the Mandena region (MAN) was moved from a forest fragment burned by human activity to a protected, though partially degraded fragment, affected by past logging and edge effect due to its small size [46]. Previously published data indicate that the animals increased body mass on average 15% and their reproductive rate did not differ compared to populations of collared lemurs living in intact habitats. However, the low average group size observed in MAN after the translocation might represent a strategy to reduce feeding competition [47].

Here, we want to assess whether and how MAN collared lemurs modified their group size, activity budget, diet, and habitat use as a response to habitat degradation. To achieve our goal, we compared the behavioral ecology of MAN groups with data previously collected on lemur groups in the more intact habitat of Ste Luce (STL), a large forest fragment 20 km north of MAN. We predict that:

1. MAN lemurs reduce intra-group feeding competition by maintaining a group size smaller than that observed in intact forests.
2. MAN lemurs modify their time-budget by increasing feeding and moving effort because of the lower density and quality of food resource in the degraded habitat. Alternatively, resting in MAN lemurs may be increased in order to save energy.
3. We also predict that the diet of MAN lemurs is nutritionally poorer and has a higher representation of fall-back species due to the expected lower food availability in the degraded forest.
4. Finally, we expect the animals in MAN to modify their habitat use by increasing ranging areas and/or number of feeding trees as a response to increased difficulties in fulfilling nutritional requirements.

## Materials and Methods

### Ethics Statement

This study was conducted with the authorization of the Commission Tripartite of the Direction des Eaux et Forêts de Madagascar (Autorisation de recherche #023 MINENVEF/SG/DGEF/DPB/SCBLF/RECH ) and the University of Pisa (Animal Care and Use Board). In accordance with the recommendations of Weatherall report, trapping of the lemurs was conducted entirely under anesthesia using a hypnotic (5 mg/kg of ketamine hydrochloride or tiletamine hydrochloride), so that the animals would not suffer/recall the capture process. Captures were carried out by an experienced Malagasy technician, Enafa Efitroaromy, via a blow-pipe darting. All animals recovered from anesthesia within 1.5 hours and were not moved from the capture area nor kept in a cage, but were followed until regaining full mobility. There were no injuries as a consequence of the captures.

### Study Sites and Species

**Figure 1:**
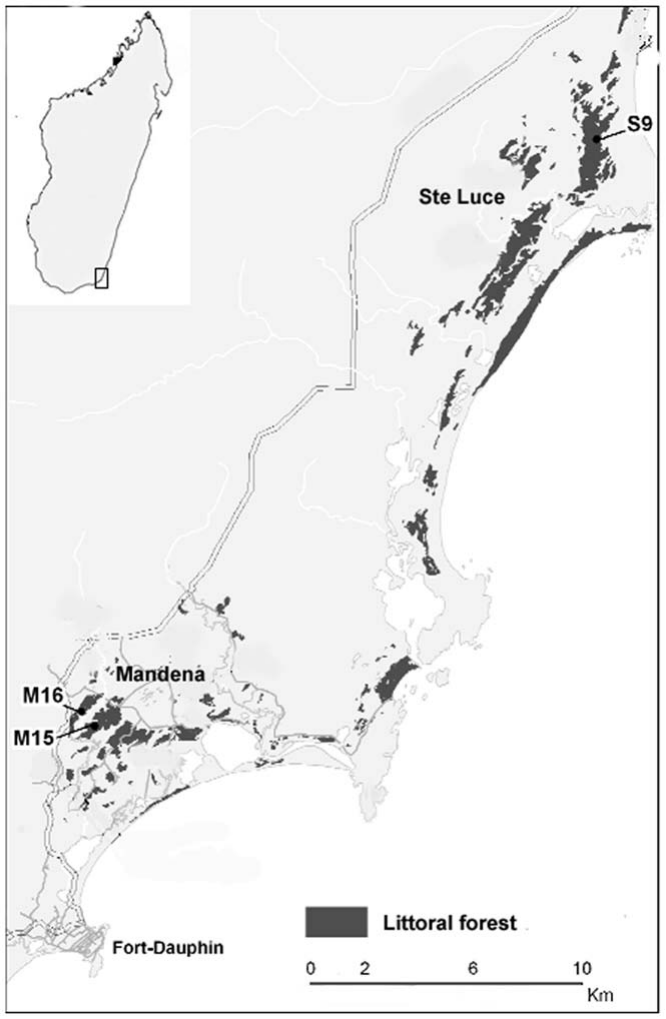
Location of sites. Study forest fragments are numbered (modified from [47]). North is up.

This comparative study was conducted in the littoral forests of MAN and STL near Fort Dauphin in south-eastern Madagascar (Figure 1). Data were first collected in STL in 2000 (fragment S9) and then in MAN in 2004 and 2007 (fragments M15 and M16) (fragment numbering system proceeds from East to West). This region is characterized by a tropical wet climate, with average monthly temperatures of 23°C (range: 18.2–25.9; n = 30), annual rainfall ranging from 1600–2480 mm, and no clear dry season [48].

The conservation zone of MAN, 11 km North-West of Fort Dauphin (24°95'S 46°99'E) is located on sandy soils at an altitude 0–20 m above the sea level [49]. The two largest forest fragments in MAN, M15 and M16, cover an area of 148 hectares of degraded littoral forest [50]. Approximately 82 ha of interspersed marsh and swamp connect the two fragments. Because collared lemurs used the swamp for travelling, feeding and resting, we considered these two forest fragments as a single area in this study. M15/M16 are the only two forest fragments where collared lemurs are still present at this site [47]. The average canopy height is 8.9±4.4 m and the understorey is dense [46]. In addition to *E. collaris*, four nocturnal (*Microcebus murinus, Cheirogaleus medius, Cheirogaleus major, Avahi laniger*), and one cathemeral lemur species (*Hapalemur meridionalis*) are found in this area.

The protected forests of STL, around 30 km north of Fort Dauphin (24°45'S 47°11'E), are among the most intact littoral ecosystems in Madagascar and possess a very high vegetation diversity [51]. The 377 ha forest block S9 is one of two fragments where collared lemurs still occur in the STL area [38]. The average canopy height is 14.7±4.3 m with a clearly stratified structure [46]. In addition to *E. collaris*, four lemur species (*Microcebus rufus, Cheirogaleus medius, C. major, Avahi laniger*) are found in this area.

Floristically MAN and STL littoral forests are very similar, suggesting that these two areas were once connected [46]. However, structural differences indicate that at the time of study, the forests of MAN represent degraded forms of the vegetation type in STL [46]. This deduction is also suggested by the disappearance of some tree families known to be logged in MAN but not in STL [46]. Forest degradation was evaluated in the two areas by estimating the percentage of surface area occupied by the canopy. This analysis resulted in the two categories of “intact to slightly degraded” and “degraded to highly degraded” for S9 and M15/M16, respectively [49] .

Phenological records from the region [48] show that there is a distinct peak in fruit production during the hot-wet season (December-February), while fruit availability is particularly low during the cool-wet season (June-August).

Collared lemurs are arboreal strepsirrhines living in multi-male, multi-female groups [38]. Mean body mass is 2.15±0.25 kg and mean body length is 46.1±2.6 cm (n = 11). Median group size in intact littoral forest is 7 (range: 2–17; n = 13) [38] and in intact rainforest is 5 (range: 2–7; n = 11) [Johnson, pers. comm.]. This lemur species is cathemeral and its dietary regime is mainly frugivorous [38].

### Census Data

In order to record group size variations, the total population of *E. collaris* in MAN was counted by complete censuses in 2004 and 2007. For this exercise, 20 people spaced at 10 m intervals, spanning the width of the M15/M16 forest, walked the entire length of the forest. Surveys usually took one day. In STL, we estimated average group size via line transects [52]. Existing trails that ran in parallel were used as transects when possible to minimize disturbance of the forest. A pair of observers walked four transects (range: 1.5–2.2 km) at a rate of 1 km per hour between the hours of 5am–7am or 4pm–6pm, stopping briefly to scan the forest for indicators of lemur presence. Twelve days per month were spent conducting systematic line transect surveys. A contact time with primate groups of 10 minutes was targeted during line transect censuses.

### Behavioral Data

Diurnal ethological data were collected on five *E.collaris* groups with different size, 3 in MAN and 2 in STL (Table 1). In MAN data were collected from May to December 2004 and from August to November 2007, while in STL from December 1999 to February 2001. Given the different time window of data collection, to allow comparisons in STL we limited the analyses to the same months when the animals were followed in MAN. Moreover, since nocturnal observations were not possible in MAN, the analysis was limited to the diurnal phase. Overall, 782 observation hours in MAN were compared with 916 hours in STL.

**Table 1:** Site, observation period, and composition of each group.

	Mandena			Ste Luce	
	Group A	Group B	Group C	Group A	Group B
Month/Year of observation	May-Dec 2004 Aug-Dec 2007	May-Dec 2004 Aug-Dec 2007	May-Dec 2004 Aug-Dec 2007	May-Dec 2000	May-Dec 2000
Adult females	1–2	2	1	3	1
Adult males	2	1–2	2	5	2
Sub-adults	0–1	0–1	0	0–2	0–1
Juveniles/Infants	0–1	0–1	0	0–5	0–2
**Total**	**4**–**5**	**3**–**5**	**3**	**8**–**13**	**4**–**6**

A total of 3 days per month was spent with each group. Each day of observation consisted of 12 consecutive hours of data collection from 6am to 6pm. Individual identification of each study animal was made using nylon collars and colored pendants, and one individual per group was radio-collared. Behavioral data were collected by the instantaneous sampling method with a 5- minute interval [53]. Focal animals were chosen from adult individuals in both study groups, and were rotated every 3 hours, so that all adult group members were evenly sampled at the end of 3 observation days (12 observation hours/day). Instantaneous data collected consisted of animal activity, food type, feeding and resting trees. Activities included feeding (food ingestion), foraging (food exploration), resting, moving, social, and other activities. Food types were noted as fruits, unripe fruits, leaves, young leaves, flowers, invertebrates, and other (bark, stems, roots, mushrooms, decayed wood). Differentiation between unripe/ripe fruits and mature/young leaves was based on differences in color, size, and texture. We estimated lemur diet by using the proportion of feeding records, as the poor visibility conditions in dense littoral forests precluded a reliable quantification of the absolute amount of food items consumed. Although temporal measures of diet may produce significant distortions of actual food intake [54], [55], since we focus on the relative proportion of food items between the two forests and not on the absolute quantification of food consumed, this method can be considered adequate for our purposes.

The Shannon index was used to determine the dietary diversity of each population and calculated using the formula: 
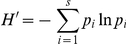
where s is the number of species consumed, p_i_ is the relative abundance of each species in the diet (records spent feeding on species i over the total feeding records). The greater the dietary diversity, the greater is *H'*. This measure is particularly useful when comparing similar dietary regimes, as it considers both the number of food species and their evenness in the diet.

### Habitat Use

All feeding trees (i.e. trees where animals were observed feeding at least in one instantaneous record) were marked with a flag and numbered to be found on a subsequent day. After behavioral data were collected, the observer returned to the trees with the help of an assistant to identify the species and to record diameter at breast height (DBH). DBH has been shown to be one of the most accurate proxies to estimate fruit production of trees and has low inter-observer variability [9]. The latitudinal and longitudinal coordinates of the feeding trees were recorded with a GPS and used to determine the size of the ranging area via the minimum convex polygon method performed after having loaded the data in the software RANGES VII.

### Nutritional Analyses

Biochemical analyses on food items eaten by the two lemur populations were conducted at the Department of Animal Ecology and Conservation of the Hamburg University in 2001 (for STL samples) and 2005–2007 (for MAN samples). Food samples were weighed with an electronic balance (fresh weight), dried in an oven for a standard period, weighed again (dry weight), ground and dried again at 50–60°C before the analyses. The lipid content was determined by extraction using petroleum ether, followed by evaporation of the solvent. Soluble proteins were assessed by BioRad after extraction of the plant material with 0.1 N NaOH for 15 h at room temperature. Soluble carbohydrates and procyanidin (condensed) tannins were extracted with 50% methanol. Concentrations of soluble sugars were determined as the equivalent of galactose after acid hydrolisation of the 50% methanol extract. Samples were analysed for neutral (NDF) and acid (ADF) detergent fibers. NDF represents all the insoluble fiber (cellulose, hemicellulose and lignin), partly digestible in species with hindgut fermentation. ADF represents the fiber fraction containing cellulose and lignin, which are mostly indigestible for *Eulemur* spp. Polyphenolic concentration was estimated as equivalents to pyrogallic acid units. A detailed review of the procedures and their biological relevance is provided by [56].

We performed separate nutritional comparisons for fruits and leaves/flowers, due to the expected different contents between these food categories. Additionally, since collared lemurs are mainly frugivorous, in order to focus on potential differences between primary and marginal fruits we compared separately species on which the animals spent at least 1% of their feeding time (primary) and the rest of the sample (marginal). Conversely, since leaves and flowers are used marginally in terms of feeding time, we included in that comparison all items eaten during the study period.

### Data Analysis

Because of the small sample size and severe deviation from normality, we used the nonparametric Mann-Whitney test to evaluate the differences between median group size recorded at the two sites in 2000, 2004, and 2007. The records of the different activities were weighted by the total number of instantaneous records. Daily average activity frequencies were calculated for each animal during the day. Then, data were pooled by month, and daily grand means per month were obtained at each site. A one-way ANOVA was used to evaluate differences between the two study sites in terms of food nutritional contents. To account for the differences between the two study sites (MAN versus STL), after having controlled for the effect of group size, we used a one-way ANCOVA entering site as independent factor and group size as covariate [57]. For the covariate we used monthly group size after log transformation in order to improve linearity for the regression and then we tested for normality via the Kolmogorov-Smirnov nonparametric test. Units of analysis for the dependent variable were monthly proportions of different activities, monthly proportion of time spent eating different food categories, monthly dietary diversity, monthly ranging areas, monthly average of the daily number of feeding trees, monthly average DBH of feeding trees. Dependent variables were also log-transformed both for the ANOVA and for the ANCOVA. We performed all tests with STATISTICA for Windows, version 6.0 and we considered p<0.05 as the significant level.

## Results

### Group Size

**Figure 2:**
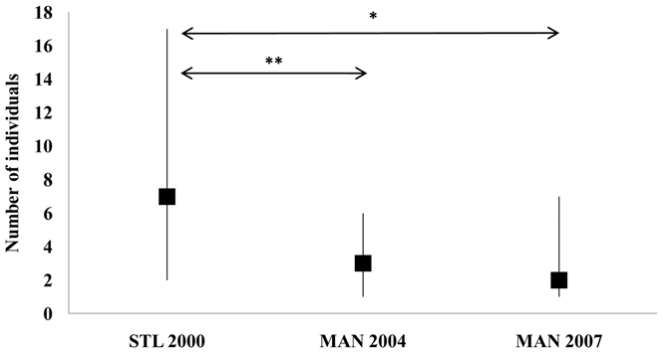
Group size at the two study sites during the three years of data recording. Values are medians and ranges. STL: Sainte Luce; MAN: Mandena. * p<.05; ** p<.001.

Average group size was larger in STL in 2000 (median: 7, range: 2–17, n = 13 groups) than in MAN both in 2004 (median: 3, range: 1–6; n = 11 groups; U = 11, p<0.001) and in 2007 (median: 2, range:1–7; n = 5 groups; U = 7.5, p = 0.010) (Figure 2). Since group composition changed over the study period, data from MAN in 2004 and 2007 were analyzed separately.

### Time-Budget

**Figure 3:**
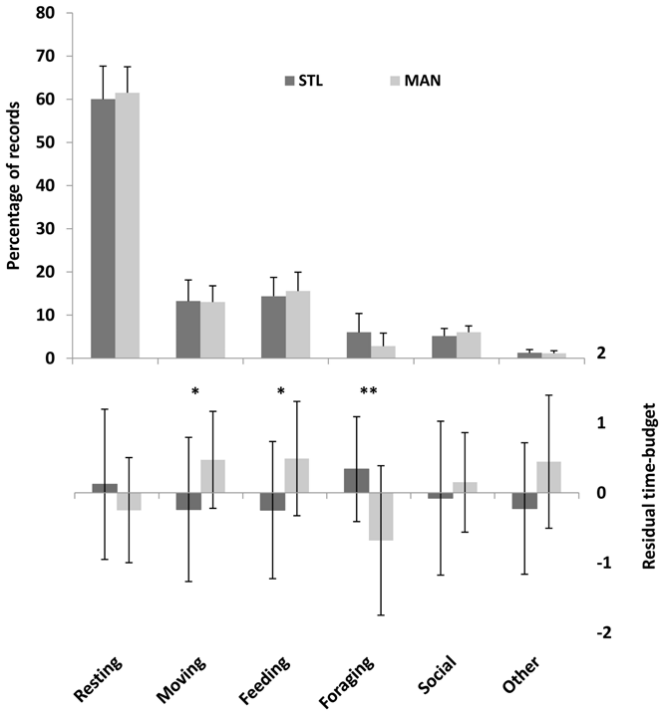
Time-budget of collared lemurs at the two study sites. Upper quadrant: monthly percentages of instantaneous records. Lower quadrant: residuals of log-transformed time budget controlling for log-transformed group size. Values are means and standard errors. STL: Sainte Luce; MAN: Mandena; * p<.05; ** p<.001.

Both in STL and in MAN, resting occupied most of the time (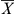
±SE: 60.8±1.1%), followed by feeding (14.9±0.8%), moving (13.1±0.1%), social activities (5.6±0.6%), foraging (4.4±2.3%), and other activities (1.2±0.1%) (Figure 3). MAN groups moved significantly more than STL groups (Site effect: F_1,21_ = 4.566, p = 0.044), once we accounted for the effect of large groups to move more than small groups (Group effect: F_1,21_ = 12.403, p = 0.002). MAN groups fed significantly more than STL groups (Site effect: F_1,21_ = 5.014, p = 0.036), once we accounted for the effect of large groups feeding more than small groups (Group effect: F_1,21_ = 8.293, p = 0.009). Also, STL groups foraged significantly more than MAN groups (Site effect: F_1,21_ = 12.307, p = 0.002). Though resting and other did not differ between the two sites, small groups rested more and performed in other activities less than large groups (Group effect: F_1,21_ = 7.601, p = 0.012 for resting; Group effect: F_1,21_ = 14.892, p<0.001 for other) (Table 2).

**Table 2:** Effects of site and group size on lemur time-budget and diet according to one-way analyses of covariance.

Time-budget	SITE	Group size
Resting	1.069	7.601*
Moving	4.566*	12.403**
Feeding	5.014*	8.293*
Foraging	12.307**	4.140
Social	0.387	0.677
Other	3.969	14.89**

Diet	Site	Group Size
Ripe fruits	0.886	0.268
Unripe fruits	1.572	0.512
Flowers	0.009	1.759
Mature leaves	6.690*	0.144
Young leaves	0.338	0.416
Invertebrates	1.264	0.003
Other	1.125	0.005

Analyses were performed on log-transformed data. Values are F-values.

*p<0.05.

**p<0.01.

### Diet

**Figure 4:**
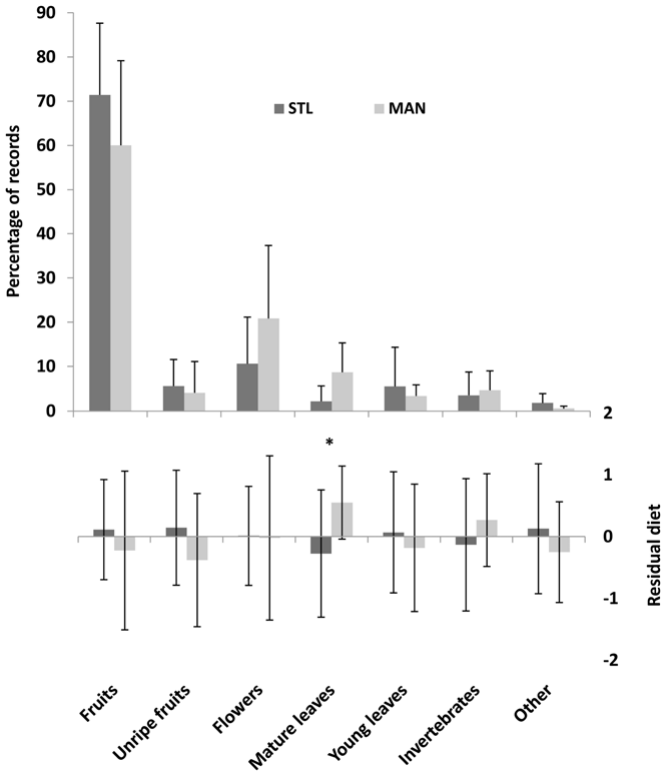
Time spent by collared lemurs eating on the various food categories at the two study sites. Upper quadrant: monthly percentages of feeding records. Lower quadrant: residuals of log-transformed feeding records controlling for log-transformed group size. Values are means and standard errors. STL: Sainte Luce; MAN: Mandena; * p<.05.

Collared lemurs were mainly frugivorous (ripe fruits 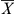
±SE: 65.7±8.1%; unripe fruits: 4.8±1.1% of total feeding time) at the two sites during the study periods, complementing their diet with flowers (15.7±7.2%), leaves (mature leaves: 5.4±4.5%; young leaves: 4.4±1.5%), invertebrates (4.0±0.8%), and other items (1.2±0.9%) (Figure 4). However, MAN lemurs spent significantly more time eating mature leaves than STL animals (Site effect: F_1,21_ = 6.690, p = 0.017) (Table 2).

Collared lemurs in STL fed on a total of 75 plant species during the study period while MAN animals used 64 species. Preferred plant species (used for more than 1% of feeding time) accounted for 71% and 89% of feeding time for STL and MAN groups, respectively (Table 3). Monthly dietary diversity evaluated by the Shannon index (*H'*, 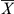
±SD: 2.16±0.59 in MAN and 1.78±0.29 in STL) did not differ significantly between sites (Site effect: F_1,21_ = 2.374, p = 0.138) and there was no influence of group size (group effect: F_1,21_ = 0.117, p = 0.736).

**Table 3:** Scientific name, family, part eaten (frm: ripe fruits, fru: unripe fruits, flo: flowers, yle: young leaves, ml: mature leaves), percentage in the diet (%), i.e. percentage of time spent by collared lemurs at the two sites feeding on the plants visited during >1% of total feeding time.

Genus and species	Family	Part eaten	% in diet
**Mandena**			
*Uapaca ferruginea*	Euphorbiaceae	frm,fru	27.8
*Canarium boivinii*	Burseraceae	frm	7.1
*Cynometra cloiselii*	Fabaceae	flo,frm,yle	6.6
*Acanthostyla aff. Longistylus*	Pandanaceae	frm, yle	5.6
*Ravenala madagascariensis*	Streliziaceae	flo	4.8
*Brexia madagascariensis*	Celastraceae	flo	4.8
*Uapaca littoralis*	Euphorbiaceae	frm,fru	4.5
*Sarcolaena multiflora*	Sarcolaenaceae	fru,flo	4.1
*Canthium* sp.	Rubiaceae	frm,fru	3.8
*Drypetes madagascariensis*	Euphorbiaceae	frm	3.0
*Vitex bracteata*	Lamiaceae	frm	2.6
*Vepris elliotii*	Rutaceae	frm,fru	2.3
*Dichaepetalium* sp.	Dichaepetaliaceae	yle, ml	2.1
*Eugenia* sp.	Myrtaceae	frm,fru	1.8
*Mammea sessiliflora*	Clusiaceae	frm	1.6
*Dillenia triquetra*	Dilleniaceae	frm, yle, ml	1.6
*Pandanus dauphinensis*	Pandanaceae	frm	1.4
*Anthocleista longifolia*	Gentianaceae	frm	1.2
*Ludia* sp.	Salicaceae	frm	1.0
**Ste Luce**			
*Syzigium* sp.	Myrtaceae	frm	20.0
*Uapaca ferruginea*	Euphorbiaceae	frm,fru	9.8
*Cynometra cloiselii*	Fabaceae	flo,frm,yle	7.7
*Olea* sp.	Oleaceae	frm	5.3
*Vepris elliotii*	Rutaceae	frm,fru	5.1
*Pandanus dauphinensis*	Pandanaceae	frm	4.9
*Eugenia* sp.	Myrtaceae	frm,fru	4.4
*Uapaca littoralis*	Euphorbiaceae	frm,fru	3.5
*Cinnamosna madagascariensis var. namorensis*	Canellaceae	frm	2.0
*Canthium variistipule*	Rubiaceae	frm,fru,yle	2.0
*Acanthostyla aff. longistylus*	Pandanaceae	frm	1.4
*Tambourissa purpurea*	Monimiaceae	frm	1.3
*Homalium albiflorum*	Flacourtiaceae	flo	1.1
*Ocotea* sp.	Lauraceae	frm	1.0

### Nutritional Content of Food

Fruits eaten during more than 1% of feeding time were considered “primary”, those eaten for less than 1% of the time were classified as “marginal”. Due to the small sample size, this distinction was not possible for flowers and leaves.

Nutritional analyses indicate that primary fruits in STL contained a significantly greater proportion of carbohydrates and a lower proportion of lipids as compared to MAN fruits. In contrast, marginal fruits in MAN contained a significantly greater proportion of tannins, fibers, and lipids, and a lower proportion of polyphenolics when compared to STL fruits (Table 4).

**Table 4:** Phytochemical characteristics as average percentage of dry matter of primary fruits (>1% feeding records), marginal fruits (<1% feeding records), and leaves/flowers eaten by collared lemurs.

		Lipids	Proteins	Sugars	P.Phenolics	Tannins	NDF-fibers	ADF-fibers
	**MAN**	**5.04**	**2.04**	**8.59**	**0.67**	**0.80**	**41.78**	**26.83**
	(14)	3.78–7.09	1.64–2.59	4.34–13.96	0.45–2.02	0.62–0.94	38.31–62.49	22.37–47.06
***PRIMARY***								
***FRUITS***	**STL**	**2.69**	**3.74**	**18.48**	**1.60**	**0.18**	**42.98**	**31.04**
	(15)	1.71–4.91	1.84–4.25	7.49–35.67	0.85–3.21	0.00–1.07	30.91–49.11	22.99–37.81
	F	4.691*	2.097	4.413*	1.316	0.399	0.238	0.064
	**MAN**	**4.02**	**2.53**	**10.78**	**0.59**	**0.84**	**44.25**	**29.42**
***MARGINAL***	(31)	2.33–9.63	1.75–3.48	3.98–16.77	0.44–0.87	0.64–1.14	38.62–53.29	20.58–38.27
***FRUITS***								
	**STL**	**2.54**	**3.24**	**15.18**	**1.74**	**0.17**	**33.26**	**21.71**
	(32)	1.29–3.78	1.95–5.21	5.56–32.21	0.94–3.10	0.00–0.36	23.33–48.94	16.85–34.97
	F	5.653*	2.137	2.153	21.781**	23.895**	5.452*	2.382
	**MAN**	**2.09**	**3.10**	**4.66**	**1.65**	**0.97**	**43.22**	**29.10**
***LEAVES***	(18)	1.64–3.09	0.86–3.75	2.50–6.48	0.73–3.68	0.68–1.12	33.51–61.15	24.28–40.50
***&***								
***FLOWERS***	**STL**	**2.35**	**3.34**	**9.33**	**2.45**	**0.24**	**33.22**	**23.07**
	(24)	1.34–4.61	1.79–6.02	6.87–15.79	1.37–4.27	0.00–1.01	25.70–41.46	16.29–29.12
	F	0.030	0.687	13.062**	1.138	1.814	5.718*	4.965*

P.Phenolics: polyphenolics are indicated as units per 100 gr of dry matter. MAN: Mandena; STL: Sainte Luce. Values are medians (in bold) and quartiles. (n) is the sample size. Statistics are F values based on one-way ANOVA on log-transformed data.

*p<.05.

**p<.01.

As for leaves and flowers consumed, these items in STL contained a significantly greater proportion of carbohydrates and a lower proportion of fibers (both NDF and ADF) than in MAN (Table 4).

### Habitat Use

MAN groups used monthly ranging areas larger than those used by STL groups (Site effect: F_1,21_ = 9.606, p = 0.005) after controlling for the effect of large groups to use larger areas (Group effect: F_1,21_ = 10.201, p = 0.004). Mean monthly ranging areas were (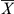
±SD) 28.11±13.28 ha in MAN and 21.15±9.41 ha in STL.

Additionally, MAN lemurs used a significantly higher number of feeding trees per day (Site effect: F_1,21_ = 10.475, p = 0.004), after controlling for the effect of large groups to use more feeding trees (Group effect: F_1,21_ = 8.716, p = 0.008). The mean daily number of feeding trees was (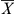
±SE) 14.25±3.06 in MAN and 11.91±3.83 in STL.

A total of 734 feeding trees used by the lemur groups in MAN and 1423 in STL were marked and measured. The analysis of the size of feeding trees showed that MAN groups fed on significantly smaller plants, in terms of DBH, as compared to STL groups (Site effect: F_1,21_ = 6.065, p = 0.023), while group size had no effect on the size of feeding trees (Group effect: F_1,21_ = 0.076, p = 0.785). The mean monthly DBH of the feeding trees used by the collared lemurs was (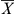
±SE) 16.60±3.81 cm in MAN and 21.02±2.78 cm in STL.

## Discussion

In the littoral forest fragments of south-eastern Madagascar collared lemurs exhibited a high degree of social and ecological flexibility. In the degraded area, lemurs were able to modify group size and several aspects of their feeding strategies, by increasing moving and feeding time, ranging areas, and number of feeding trees. Considering that body mass did not differ between the two areas [47], the above strategies were apparently able to counteract a reduction in both nutritional quality of food items and size of feeding trees in MAN. Our findings are in line with other studies on lemurs that show a relative tolerance to a certain degree of habitat degradation and fragmentation [44], [45], [25], [13], [50]. However, these results contain relevant implications and potential recommendations for collared lemur conservation in rainforest habitats. First, when living in or moved to degraded habitats these lemurs require much larger ranging areas than in intact or semi-intact forests. Second, in order to counteract intra-group competition they split into small groups with potential effects for demographic dynamics. Though some dietary flexibility has been observed, the above phenomena seem to be the consequence of the incapability of these lemurs to shift to a more folivorous diet, in contrast to what has been observed in other brown lemur populations [33], [58].

Our results suggest that the observed reduction in group size in the degraded MAN forest fragment compared to the more intact STL fragment was more likely a consequence of a reduction in habitat size and quality. This response is well known from studies on lemurs and other primates [40]–[44], [59], [60]. Predation pressure, the other main factor influencing group size, seems to have represented a less urgent priority for these collared lemurs. In fact, we would expect a larger group size in MAN than in STL, considering that the fossa (*Cryptoprocta ferox*), the main lemur predator, has not been reported in the latter area in two decades, while it visits MAN occasionally [47]. By the end of 2003 until 2007, some of these carnivores had been seen regularly in the forest. In 2004 only, at least four *E. collaris* were killed by *C. ferox* in MAN [47]. The disappearance of several other *E. collaris* in MAN during the study period may indicate that predation pressure was probably much higher than in STL. Small groups might also have suffered higher predation risk from large diurnal raptors, such as *Polyboroides radiatus* and *Accipiter henstii*, both present in the two areas, although attacks were rarely reported [47].

The increased feeding activity of the MAN lemurs concomitant with greater time spent feeding on leaves or less nutritious food is consistent with patterns observed on other frugivorous-folivorous primates living in fragmented/degraded areas. Howling monkeys (*Alouatta palliata*) in forest fragments visit more food sources when feeding from more leaves, which results in more traveling and feeding and less resting [3], [61], [62]. Low habitat quality is associated with increased feeding and decreased resting in baboons (*Theropithecus gelada* [63], *Papio cynocephalus* [64]), while an increased feeding on leaves or some fall-back food is also observed in guenons (*Cercopithecus cephus* [5]), macaques (*Macaca tonkeana* [65]), and lemurs (*Propithecus diadema* [66]) living in fragments.

Not surprisingly, the augmented feeding effort recorded in this study seems to have been a direct consequence of spending more time eating mature leaves and low quality food in a habitat impoverished of large fruiting trees. This hypothesis is well supported in our case by the nutritional analyses of all food items, both fruits and leaves/flowers, which showed a drop of carbohydrates and, thus, of available energy for MAN lemurs. Thus, the strategy followed by collared lemurs may have been an increase of feeding efforts in response to a decrease in food energy. This tactic is not a paramount behavioral response, however, and site-specific factors may lead to different choices, even when looking at the same species. Indeed, time-budget differences were not observed in other primates (*A. palliata* [67], [68], *Colobus guereza* [11]) when logged and continuous forests are compared. The opposite strategy has also been observed, with decreased feeding and increased resting in other *C. guereza* groups [69], in *Procolobus rufomitratus* [70], and in *Macaca silenus* [71] living in fragmented forests.

Interestingly, another factor possibly accounting for the difference in feeding efforts between MAN and STL may have been the processing time of different food items. We could not collect specific data on processing time, but the top food item in MAN were the drupes of *Uapaca ferruginea*, which need to be opened to swallow the pulp discarding the husk. Conversely, the top food item in STL were the small berries of *Syzigium* sp., easily and quickly swallowed without processing. Food processing time is an aspect which deserves further investigation when comparing lemur time-budgets.

Increased feeding on mature leaves, as observed in our lemurs, may also require a greater effort to meet specific nutritional requirements [72] and to avoid an overload of toxins or digestibility reducing compounds [73], [74]. This fact would be particularly important for animals with no adaptations for a strictly folivorous diet, such as for *Eulemur* species [75]. We do not have data on presence/dosage of secondary compounds, tannins and polyphenolics excepted, but an overall tendency for a higher content of the former and a lower content of the latter was observed in MAN food items. These differences were significant in marginal fruits. Tannins, in particular, are known to bind proteins and reduce their digestibility [76], therefore limiting tannin ingestion might be necessary for MAN lemurs, since their food was extremely poor in proteins (see table 4). The need to limit tannin ingestion may also have restricted the possibility for a more dramatic shift to leaves in our lemurs, since in rainforests leaves are known to contain more secondary compounds and lower protein to fiber ratios, due to their long life-span [77], [78]. As a matter of fact, *Eulemur* species in western deciduous forests have been observed to shift to a folivorous diet during some periods of the year [58], [79], [80] or even year-round [33], which does not seem to be an option for their congenerics in the eastern rainforests [26], [32], [38]. This seems to be the case for our collared lemurs, as the overall time spent feeding on leaves was relatively low in both study areas, though significantly greater in the degraded fragment. Avoiding tannins may be important also when lemurs remain mainly frugivorous, considering the high tannin content and low protein content of the marginal fruits eaten by the MAN lemurs. In support of our findings, a low fruit protein content seems to be a general rule in Madagascar as compared to other continents [81].

The analysis indicated that, once the effect of group size was removed, collared lemurs in MAN visited more and smaller food trees than those living in the intact forest of STL, which, in turn, resulted in increased ranging areas. This phenomenon may be linked to the fact that MAN groups were not able to feed from large trees, which generally contain more resources and are depleted more slowly [9]. Large trees are in fact the most vulnerable to fragmentation, forest degradation [7], and human exploitation [4]. The loss of the largest food patches is a general syndrome faced by primates in fragmented forests [1], [3], [58], [82]–[84], and it might have been the driving force for the observed group size reduction in MAN.

Group fission as a response to changes in food patch size has already been recorded for a number of lemur species [41]–[44]. In this respect, *Eulemur* species are not an exception [40]. In particular, a 46% decline in average group size of *E. f. rufus* has been associated with decreased fruit availability in Ranomafana [26]. However, long-term data on this genus from continuous rainforests indicate that habitat shifting is the main response of *Eulemur* species to severe food scarcity [26], [32], [85]. In Ranomafana *E. f. rufus* are the only lemurs known to migrate up to 5 km away from their home-ranges when fruits are scarce in their habitat [26]. This strategy theoretically allows groups to remain cohesive while they are forced to range further in search for food [23]. Although real migrations have not yet been observed in collared lemurs, the STL largest group was able to expand considerably its monthly ranging area from 23 to 56 ha during the lean season [86]. Such an option was not a choice for the lemurs living in the small MAN fragment, thus possibly forcing animals to split into small subgroups in order to reduce feeding competition. Interestingly, however, groups of lemurs were observed on several occasions to cross the savannah after their translocation into the MAN area, although it is not clear whether this behavior was a response to low food availability or a kind of homing.

Malagasy rainforests are known to naturally experience long periods of fruit scarcity [28], [48]. Additionally, fruiting tree cycles are irregular [27] and productivity is relatively low due to poor soils and erratic climate [14], [29]. This ecological scenario has set the matrix for lemur communities to evolve strategies to deal with periods of food scarcity [27], [87]. In this respect, frugivorous lemurs are expected to be more resilient to a certain degree of habitat alteration compared to their ecological equivalents in other primate communities [45], [50], [75]. Our data indicate that collared lemurs in littoral forest fragments were actually able to modify a number of aspects of their behavioral ecology. However, responses to habitat degradation may change among habitats, species, or even populations [23], [25]. Additionally, given the potential variation in food availability between years [48], our conclusions have to be taken with caution until more long-term simultaneous data will be available. Nevertheless, our results contain relevant implications for brown lemur conservation. The observed flexibility in feeding, social, and ranging patterns should be carefully considered when relocating frugivorous lemurs or when selecting suitable areas for their in situ conservation.
